# Silver (I) as DNA glue: Ag^+^-mediated guanine pairing revealed by removing Watson-Crick constraints

**DOI:** 10.1038/srep10163

**Published:** 2015-05-14

**Authors:** Steven M. Swasey, Leonardo Espinosa Leal, Olga Lopez-Acevedo, James Pavlovich, Elisabeth G. Gwinn

**Affiliations:** 1 Department of Physics, UCSB, Santa Barbara, CA 93117; 2 Department of Chemistry and Biochemistry, UCSB; 3 Department of Applied Physics, Aalto University, 00076 Aalto, Finland

## Abstract

Metal ion interactions with DNA have far-reaching implications in biochemistry and DNA nanotechnology. Ag^+^ is uniquely interesting because it binds exclusively to the bases rather than the backbone of DNA, without the toxicity of Hg^2+^. In contrast to prior studies of Ag^+^ incorporation into double-stranded DNA, we remove the constraints of Watson-Crick pairing by focusing on homo-base DNA oligomers of the canonical bases. High resolution electro-spray ionization mass spectrometry reveals an unanticipated Ag^+^-mediated pairing of guanine homo-base strands, with higher stability than canonical guanine-cytosine pairing. By exploring unrestricted binding geometries, quantum chemical calculations find that Ag^+^ bridges between non-canonical sites on guanine bases. Circular dichroism spectroscopy shows that the Ag^+^-mediated structuring of guanine homobase strands persists to at least 90 °C under conditions for which canonical guanine-cytosine duplexes melt below 20 °C. These findings are promising for DNA nanotechnology and metal-ion based biomedical science.

The long-standing biochemical interest in metal-DNA interactions now extends into the field of DNA nanotechnology^1^, where incorporation of strongly bound metal cations promises to realize more robust, diversely functional DNA-based materials^2^,^3^,^4^,^5^. This potential stimulated the recent development of artificial bases that form metal-mediated pairs bridged by Ag^+^, Cu^2+^ and Hg^2+^ 6,7,8,9. The identification of more diverse metal cation pairings of the natural bases could remove the need to incorporate expensive synthetic elements and amplify the impact of such metal-mediated pairings. Ag^+^ is uniquely interesting because it exhibits unusually specific interactions with DNA, binding exclusively to natural bases rather than the negatively charged phosphate backbone (Hg^2+^ also associates exclusively with the bases, but is far more toxic)^5^. The potential that Ag^+^-DNA interactions hold for nanotechnology is already exemplified by the fluorescent, DNA-stabilized silver clusters^10^,^11^ used recently in novel chemical and biochemical sensing schemes^12^. These nano-optical, DNA based materials are known to incorporate Ag^+^ as well as neutral silver atoms^13^, indicating that Ag^+^-DNA interactions are key to stabilizing the fluorescent clusters. In addition to the previously known bridging of cytosine (C) bases by Ag^+^ 14,15, the formation of fluorescent silver clusters on homo-base guanine (G) strands of DNA and RNA^16^ and the Ag^+^ induced dimerization of individual modified guanine bases in non-aqueous solution^17^ suggests that Ag^+^ might also bridge G bases in DNA oligomers. In biochemistry, the strong interactions of G bases with Pt^2+^ are thought to be key to important chemotherapy drugs^18^,^19^. Thus the discovery of stable Ag^+^-G binding might find use in treatment of disease associated with mutations. Ag^+^-base interactions may also underlie the antimicrobial action of silver nanoparticles^20^. The known biochemical roles of metal cations suggest that a better understanding of how Ag^+^ binds to the natural DNA bases could aid future development of disease therapeutics.

Despite this compelling potential, surprisingly little is known about how Ag^+^ interacts with the natural bases when DNA is not conformationally constrained by the canonical Watson-Crick (WC) hydrogen bonding of adenine (A) to thymine (T) and C to G. Early studies of biological double-stranded (ds) DNA could not unravel the diversity of base-Ag^+^ interactions due to the mixed A, C, G and T composition^21^,^22^,^23^,^24^,^25^,^26^,^27^. More recent studies of synthetic dsDNA used the insertion of single-base mismatches to examine which bases could be bridged by Ag^+^, when subject to the conformational constraints of the WC-paired surroundings^14^,^28^,^29^. In addition to C-Ag^+^-C, evidence for a C-Ag^+^-A pair has been reported^30^, while the recent experimental literature is conflicting on the possibility of C-Ag^+^-T pairing^28^,^29^, and mostly silent regarding Ag^+^ interactions with G^31^. Prior computational studies of Ag^+^-base interactions have been challenged by the plethora of possible binding geometries, especially for G^32^,^33^,^34^,^35^,^36^, and have focused largely on binding geometries compatible with WC-like structure.

Although many prior studies have focused on Ag^+^ incorporation into a canonical dsDNA environment, there is no *a priori* reason to assume that base pairing by hydrogen bonding will persist in the presence of Ag^+^. The dominant mode of binding must depend on the affinities and geometries of Ag^+^-base interactions relative to WC pairing. Here we remove the constraint of WC pairing by focusing on homo-base deoxyoligonucleotides and mixtures of these A_N_, C_N_, G_N_ and T_N_ strands, for strand lengths N = 6 to N = 20 bases (Fig. 1a), at neutral pH. We use relative abundances in electrospray ion mass spectrometry (ESI-MS) to determine the ordering of Ag^+^ affinities to each homo-base strand. The competition of Ag^+^-mediated base pairing with WC pairing is tested by studies of the mixed complementary strands. Through quantum chemical calculations that explore an unrestricted space of binding geometries, we find that the order of binding energies (BE) for the most stable Ag^+^-bridged homobase complexes agree with abundance trends in ESI-MS data. Strikingly, experiments show that G_N_ strands form fully Ag^+^-bridged duplexes, G_N_-(Ag^+^)_N_-G_N_, that are more stable than the WC paired C_N_-G_N_ duplex.

## Results and Discussion

### Detection of (Ag^+^)_N_-DNA products by ESI-MS

**Figure 1:**
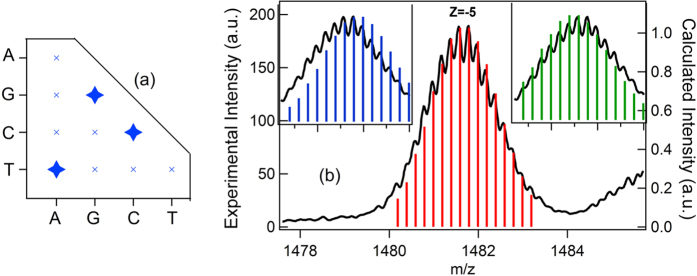
(**a**) Schematic of the homo-base strand types and combinations studied. Stars denote the detected Ag^+^-bridged duplexes. (**b**) Example of isotope peak envelope resolved in MS for C_11_-(Ag^+^)_11_-C_11_. Black lines: data. The total mass of the ionized species (m) is given by **m** = **m**_**DNA**_ **+ m**_**Ag**_**N**_**Ag**_ **– n**_**pr,**_ where m_DNA_ is the mass of the unionized DNA strand, m_Ag_N_Ag_ is the mass of the total silver content, and n_pr_ is the number of protons removed by negative mode electrospray ionization. The charge state, z (negative) of the ionized species is z = Q_Ag_/e – n_pr_, where Q_Ag_ is the total charge associated with the silver content. Bars show the calculated isotope peak patterns for a net charge on the silver content of Q_Ag_ = +10*e* (blue), +12*e* (green)(insets) and +11*e* (red) associated with the silver atom content. The best fit at a charge of +11*e* confirms that all of the attached silver atoms are cations, Ag^+^.

We use high-resolution, negative ion ESI-MS to determine the composition of complexes formed by Ag^+^ attachment to the homo-base oligomers. ESI-MS is a powerful tool for detecting non-covalently bound molecules to DNA in solution^37^ and for investigating solution binding stoichiometries to DNA^38^. Our use of high-resolution MS resolves the isotope peak envelopes (Fig. 1b), which enables determination of absolute composition, not just the ratio of silver cations per base (stoichiometry). This is important for unambiguous determination of strand dimerization by Ag^+^. By fitting the calculated isotope peak envelope to the MS data^13^,^39^,^40^, we find that all of the attached silver atoms are cationic (Ag^+^), as expected.

**Figure 2:**
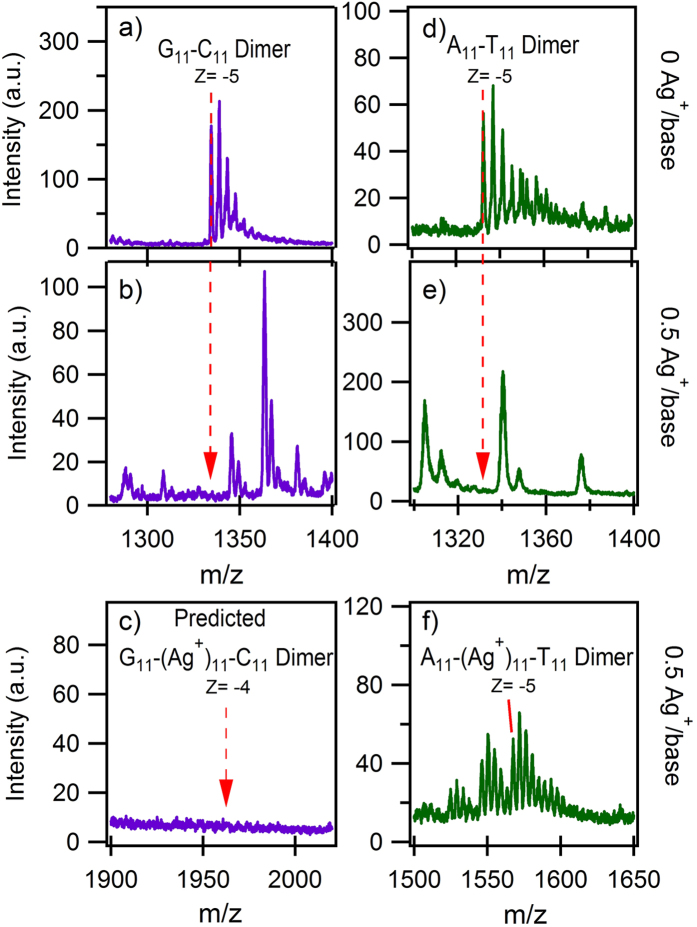
Effects of Ag^+^ on solutions of mixed C_11_ and G_11_ (**a**)-(**c**), and solutions of mixed A_11_ and T_11_ (**d**)-(**f**), at 40 μM per strand. (**a**) Mass spectra (MS) of the C_11_-G_11_ mixture at 0 Ag^+^/base and (**b**) 0.5 Ag^+^/base. Dashed line: m/z for the WC paired G_11_-C_11_ duplex, present in the absence of Ag^+^ (a) but undetectable after adding Ag^+^(b). (The peak envelope to higher m/z in (a), (d) and (f) is attachment of Na^+^ from residual salts). (**c**) No G_11_-(Ag^+^)_N_-C_11_ products were detected, exemplified by the absence of G_11_-(Ag^+^)_11_-C_11_ (expected at dashed line). (**d**) MS of A_11_-T_11_ mixture at 0 Ag^+^/base and (**e**) 0.5 Ag^+^/base. Dashed line: m/z for the WC paired product showing no detectable signal after adding Ag^+^. (**f**) A_11_-(Ag^+^)_N_-T_11_ products did form, exemplified by A_11_-(Ag^+^)_11_-T_11_ (dashed line). Additional, unlabeled peaks in (b) and (e) are various (Ag^+^)_N_-DNA products (see Fig. S1).

To investigate the possible disruption of WC pairing by Ag^+^, we combined C_11_ with G_11_, and A_11_ with T_11_, at 40 μM/strand in 10 mM ammonium acetate (pH 7). Mass spectra of the mixture of C_11_ and G_11_ strands (Fig. 2a) show the expected peaks for the WC-paired C_11_-G_11_ duplex, with additional peaks for the individual C_11_ and G_11_ strands (Fig. S1). After addition of 0.5 Ag^+^ per base, the C_11_-G_11_ duplexes entirely vanish from the mass spectra (Fig. 2b). New peaks appear for Ag^+^-decorated strand monomers, C_11_-(Ag^+^)_N_ and G_11_-(Ag^+^)_N_; and for Ag^+^-paired homoduplexes, C_11_-(Ag^+^)_N_-C_11_ and G_11_-(Ag^+^)_N_-G_11_. Strikingly, there were no detectable C_11_-(Ag^+^)_N_-G_11_ *hetero*duplex or triplex products (Fig. 2c). If present, such products are at too low concentration to produce detectable ion currents, while the Ag^+^-paired homoduplexes are present in concentrations that result in high ion currents. We infer that the binding of Ag^+^ in G_11_ or C_11_ duplexes is more stable than canonical WC pairing of G_11_ to C_11_, and also more stable than C_11_-(Ag^+^)_N_-G_11_ pairing. For A_11_-T_11_ (Fig. 2d), WC-paired strands were undetectable after addition of 0.5 Ag^+^ per base (Fig. 2e), but heteroduplex A_11_-(Ag^+^)_N_-T_11_ products were detected (Fig. 2f) as well as A_11_-(Ag^+^)_N_. There were no detectable homoduplex A_11_-(Ag^+^)_N_-A_11_ or T_11_-(Ag^+^)_N_-T_11_ products.

Measurements on every strand combination (Fig. 1a) found A_11_-(Ag^+^)_N_-T_11_ as the only detectable Ag^+^-bound heteroduplex. Apparently the favored mode of attachment of Ag^+^ to A_11_ is incompatible with homoduplex duplex formation under these solution conditions. Other, less stable Ag^+^-bridged heteroduplexes may exist but be reduced to undetectable levels by formation of C_11_-(Ag^+^)_N_-C_11_, G_11_-(Ag^+^)_N_-G_11_ and A_11_-(Ag^+^)_N_ instead.

**Figure 3:**
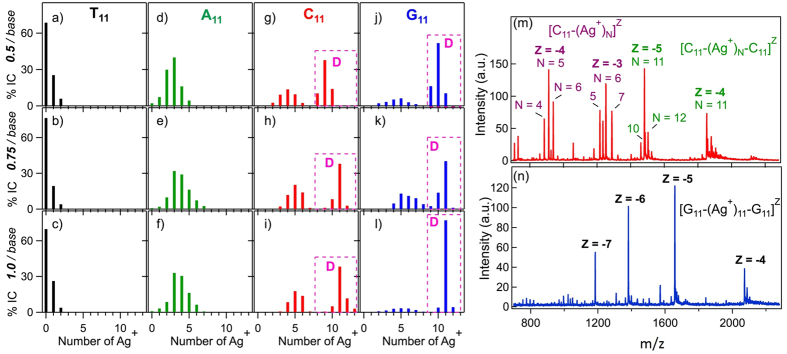
(**a-l**) Percentage of the total integrated counts (%IC) for each detected Ag^+^-bearing DNA product plotted *versus* number of attached Ag^+^. *Top, middle and bottom rows*: solutions with 0.5, 0.75 and 1 Ag^+^/base. Product yields are qualitatively different depending on base type. The boxed peaks labeled “D” are Ag^+^-paired duplexes, containing two copies of the strand. All other peaks correspond to strand monomers. Data are for the highest abundance charge state of each product. (**a-c**) T_11_ solutions exhibit the bare strand as the major product. (**d-f**) A_11_ shows a range of Ag^+^ attachment to strand monomers and no duplexes. (**g-i**) C_11_ exhibits strand monomer and duplex products. (**j-l**) G_11_ products are heavily biased to duplexes. The duplex with 11 bridging Ag^+^, G_11_-(Ag^+^)_11_-G_11_, is the overwhelming majority product at 1 Ag^+^/base (l). (**m,n**) Mass spectra of C_11_ and G_11_ solutions at 1 Ag^+^/base, corresponding to % IC plots i) and l). Major peaks are labeled.

To better understand the patterns of Ag^+^ attachment, we investigated the products formed on all individual strands at Ag^+^/base ratios of 0.5, 0.75 and 1.0. Figure 3a-l show the integrated counts (IC) measured for the highest abundance charge state, z_max_, of the strand monomer (z_max_ = −3 or −4) and duplex (z_max_  = −5 or −6) products. Figure 3m,n show full spectra for C_11_ and G_11_ at 1 Ag^+^/base (Figs. S2-S4 show all other full spectra). The IC provide a semi-quantitative comparison of the relative abundances of Ag^+^-DNA products (IC do not give quantitative product yields due to dependence of count rates on z and the possibility of unbinding events during ESI). The small shifts in Ag^+^/strand stoichiometry from 0.75 to 1.0 Ag^+^/base suggest that the silver binding to DNA is near saturation. For T_11_ (Fig. 3a–c), the dominant product at all Ag^+^/base was the bare strand. A_11_ formed a wider range of strand monomer products, with A_11_-(Ag^+^)_3_ dominant (Fig. 3d–f). It appears that A_11_ binds Ag^+^ more stably than T_11_, as expected at neutral pH^41^.

For C_11_ (Fig. 3g–i,m), dominant products at the higher Ag^+^ concentrations were C_11_-(Ag^+^)_11_-C_11_, corresponding to Ag^+^ bridging each pair of C bases, and the strand monomer product C_11_(Ag^+^)_5_. The presence of the Ag^+^-bound C_11_ duplexes (dashed boxes labeled “D”, Fig. 3g–i) agrees with previous circular dichroism (CD) studies that suggested Ag^+^-induced dimerization of a C_8_ strand^42^.

Mass spectra for G_11_ (Fig. 3j–l,n) exhibit narrower product distributions than for C_11_. The fully Ag^+^-bridged duplex, G_11_-(Ag^+^)_11_-G_11_, dominates overwhelmingly at 1 Ag^+^/base (Fig. 3l,n). Small amounts of strand monomer G_11_-(Ag^+^)_N_ products are still detectable, but in much reduced abundances relative to strand monomer Ag^+^-C_11_ products. Results were similar for C_N_ and G_N_ strands with N = 6 and 20 (Fig. S5).

The relative abundances in Fig. 3 reflect the partitioning of Ag^+^ between the solvent and the various Ag^+^-DNA products. The hydrated state of the Ag^+^ in solution is the same in all cases. If the hydrated Ag^+^ has lower free energy than the Ag^+^-DNA complexes, the bare DNA strand will be the major product. This is the case for T_11_, for which the bare strand comprises ~70% of all products. For A_11_ the bare strand is still detectable, but as only 1-2% of all products. This indicates that the hydrated Ag^+^ is no longer the thermodynamically favored state and consequently, that the complexes of Ag^+^ with A_11_ have lower free energy than the complexes of Ag^+^ with T_11_. For C_11_ and G_11_, the bare strand is undetectable, indicating a further lowering in free energy of the complexes of Ag^+^ with C_11_ and G_11_. The fully Ag^+^-bridged G_N_^-^(Ag^+^)_N_-G_N_ duplex appears to be the most stable of all Ag^+^-G_N_ complexes. The higher presence of duplex relative to monomer strand products for G_N_ than for C_N_ may indicate a reduced propensity for strand self-folding around Ag^+^ for the larger G base. To our knowledge this is the first study to detect Ag^+^-mediated pairing of guanine bases, a possibility that has not been investigated previously without the imposition of structural constraints from canonical WC pairing.

### Thermal stability of silver mediated DNA homobase duplexes

**Figure 4:**
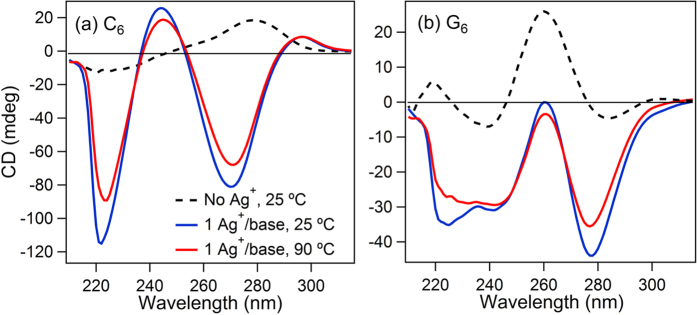
Circular dichroism (CD) spectra of C_6_ (**a**) and G_6_ (**b**). Dashed black lines: data for the bare strands at 25 °C. Blue lines: with 1 Ag^+^/base at 25 °C. Red lines: with 1 Ag^+^/base at 90 °C. The restructuring of the CD spectra upon addition of Ag^+^ reflects the reconfiguration of the DNA by incorporation of Ag^+^. Persistence of the dichroic peaks to the highest temperature accessible experimentally shows that this structural change is thermally robust.

To investigate thermal stability we carried out circular dichroism (CD) studies of C_6_ and G_6_. The solutions contained 1 Ag^+^/base (pH 7) at DNA concentrations of 17 μM. Silver mediated homoduplex products remain abundant at this concentration in mass spectra (Figure S6). In the absence of Ag^+^, the C_6_ solution (Fig 4a; black dashed line) shows the expected peak structure for predominantly unstructured cytosine oligonucleotides^43^,^44^. The peak structure of the G_6_ solution (Fig. 4b) indicates some presence of parallel G-quadruplex type structures^45^,^46^. With Ag^+^, the CD spectra are dramatically altered by base-Ag^+^ interactions (Fig. 4). Remarkably, the Ag^+^-imposed structure persists to the highest temperature we investigated, 90 °C (red curves). For comparison, the nearest-neighbor two-state model calculated melting temperature (mfold) for the C_6_-G_6_ duplex formed by canonical WC pairing is 16 °C for the low ionic conditions in Fig. 4. Apparently Ag^+^-bridging of G to G and C to C bases is much more stable than canonical WC pairing. This discovery of highly stable, silver mediated pairing of G bases, in addition to the previously known C-Ag^+^-C pairing, has promise to broaden the range of applications for DNA nanotechnology, which is currently limited by the low thermal melting temperatures imposed by Watson-Crick pairing, and may also impact development of disease therapeutics based on metal-DNA interactions.

### Quantum chemical calculations of binding strengths and geometries

For theoretical investigation of the geometries and stabilities of Ag^+^-mediated base pairing, we consider two bases and a silver atom in vacuum with the charge of the entire system set to +1. To calculate the electronic ground state we used density functional theory with a real space basis and the projector-augmented wave method^47^. The exchange correlation functional PBE+TS09 was chosen to account for van der Waals dispersion interactions^48^,^49^. The grid spacing was 0.18 Å and the calculation was spin-polarized. Per atom, the electronic configuration of the valence electrons are H(1s^1^), O(2s^2^2p^4^), C(2s^2^2p^2^), N(2s^2^2p^3^), Ag(4p^6^4d^10^5s^1^), including a scalar relativistic correction and a frozen core. We also report CAM-B3LYP binding energies (BE) calculated with the Gaussian code^50^ and a LANL2DZ/ECP basis set for the silver atom and 6-311+G(d,p) for the rest.

Starting from many initial geometries (SI), a global search is performed *via* force optimization using the Hessian matrix (BFGS algorithm in ASE) until the residual force is below 0.02 eV/Å. To verify that the relative ordering of silver-mediated BE is invariant with functional, CAM-B3LYP/6-311+G(d,p) was used. The same ordering as PBE+TS09 is obtained (SI).

**Figure 5:**
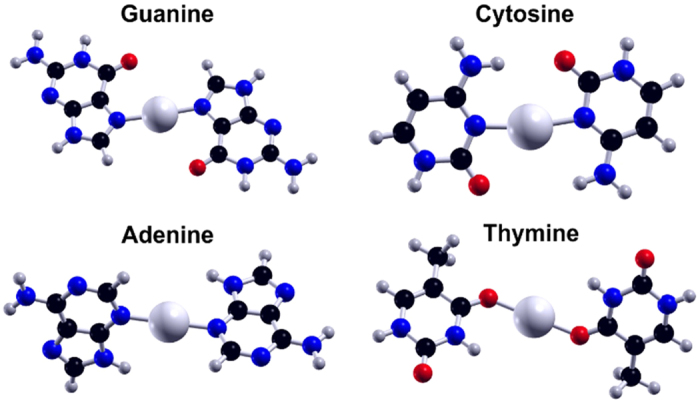
Calculated ground state geometries of Ag^+^-mediated homo-base pairs. Binding energies decrease in the order G > C > A > T (see text). G-Ag^+^-G and C-Ag^+^-C are planar, while A-Ag^+^-A and T-Ag^+^-T are non-planar.

Figure 5 shows the calculated ground state geometries for the Ag^+^-bridged bases. Bond lengths and dihedral angles are specified in the SI, along with the geometries of higher-lying structures. For homo-base pairs bridged by Ag^+^, the calculated BE, defined as the sum of the energy of fragments minus the energy of the complex, are 129.7, 126.2, 111.9, and 91.5 kcal/mol for G-Ag^+^-G, C-Ag^+^-C, A-Ag^+^-A and T-Ag^+^-T, respectively. These BE are all higher than those for attachment of Ag^+^ to the individual bases, calculated in prior work to be to be 77.08, 71.16, 60.30 and 51.19 kcal/mol for the most stable G-Ag^+^, C^-^Ag^+^, A^-^Ag^+^ and T-Ag^+^ complexes, respectively (in the most stable C-Ag^+^ and G-Ag^+^ configurations, the silver coordinates to two binding sites on one base^51^). With respect to prior work with PBE only functional, the BE is very close and ordering is the same. The pairing of bases by Ag^+^ and the attachment of Ag^+^ to individual bases show the same base-dependent ordering of BE, G ~ C > A > T.

In the absence of interfering steric factors, the higher BE for base-Ag^+^-base pairing than for individual base-Ag^+^ binding should result in higher yields of Ag^+^-bridged duplexes than strand monomer products with Ag^+^. However, experimentally we observe Ag^+^-bridged homoduplexes only for G_N_ and C_N_ (Fig. 3; dashed boxes labeled “D”). The calculated ground state structures are consistent with this experimental observation. The bases in the G-Ag^+^-G ground state are very nearly coplanar, with dihedral angle θ = 181.2°, and close to co-planar for C-Ag^+^-C (θ = 171.9°), as required to permit base-stacking interactions in Ag^+^-paired DNA strands containing multiple bases. In contrast, for A-Ag^+^-A and T-Ag^+^-T the ground state structures have highly non-coplanar bases with θ = 101.6° and 140°, respectively. Such a twisted geometry would disrupt base-stacking and sterically hinder the formation of Ag^+^-bridged strand homoduplexes of A and of T bases, consistent with the absence of these products in data (Fig. 3). We expect that the small (~10°) calculated rotation of the base planes in C-Ag^+^-C may account for the relatively high presence of strand monomer complexes, C_N_-(Ag^+^)_m_, in Fig. 3. Overall, the stoichiometric abundances of Ag^+^ bound to bases in Fig. 3 agree with the ordering of the calculated BE, G ~ C > A > T.

Our calculations find that in G-Ag^+^-G, Ag^+^ binds through the N7 atom of G, a site that does not engage in WC pairing (Fig. 5; see Fig. S7 for site numbering). This can account for the non-detection of G-Ag^+^-G pairing in prior studies of single G base mismatches within otherwise WC-paired duplexes, in which the canonically-paired surroundings restricted presentation of the mismatch bases^28^. In C-Ag^+^-C, we find that Ag^+^ bridges the N3 atom of the C bases. This site also participates in WC pairing, consistent with the observation of Ag^+^-pairing of C base mismatches in earlier work on WC duplexes^28^. For both G-Ag+-G and C-Ag+-C, hydrogen bonding helps stabilize the complexes. We note that G-Ag^+^-C has a comparable predicted BE (SI), with nearly co-planar bases. Presumably factors that are beyond the present calculations, specifically base stacking and solvation, cause the preferential formation of G_11_-(Ag^+^)_11_-G_11_ and C_11_-(Ag^+^)_11_-C_11_ rather than C_11_-(Ag^+^)_11_-G_11_ (Fig. 2).

Our calculations for binding energy (BE) contribute to the enthalpy only while unaccounted entropic costs of complex formation also contribute to relative solution abundances. For canonical WC duplexes, it is well-known that the entropic costs of duplex formation substantially compensate the enthalpic contributions to the free energy^52^,^53^; however the free energy and the enthalpy correlate, as evidenced by higher melting temperatures for canonical G-C rich duplexes compared to A-T rich duplexes. Because the BE of the Ag^+^-bridged bases are substantially higher than for canonical WC pairing (25.5 kcal/mol for G-C and 13.5 kcal-mol for A-T with PBE+TS09, respectively), we expect that the relative solution free energies of base-Ag^+^-base products will have the same ordering as the calculated BE if the calculated ground state geometries are consistent with formation of helices and the hydration free energies of the Ag^+^-bridged duplexes are similar to each other (as is the case for canonical duplexes of varying composition)^54^. For the homo-base, Ag^+^-bridged duplexes, relative experimental abundances (G ~ C > A > T) are consistent with the ordering of the calculated BE. The narrower product distribution for G-Ag^+^-G complexes (which are heavily dominated by G_11_-(Ag^+^)_11_-G_11_) than for C-Ag^+^-C complexes may reflect greater solvent stabilization associated with the higher solvent accessible area for the larger G base, and greater stacking tendencies.

For the heterobase Ag^+^ duplexes, the lowered symmetry in steric properties adds additional complexity. The data for mixed A_11_-T_11_ (Fig. 2 and S1b) show T-Ag^+^-A heterobase complexes but no T or A homobase duplexes. For T-Ag^+^-T, this is consistent with the lower calculated BE (91.5 kcal/mol) than for T-Ag^+^-A (102 kcal/mol); however for A-Ag^+^-A, the calculated BE (111.9 kcal/mol) is ~10 kcal/mol higher than for the hetero-base complex. We expect that the non-planar calculated structure of A-Ag^+^-A (SI) may be destabilizing relative to A-Ag^+^-T when stacking and hydration are included, accounting for the absence of A-Ag^+^-A in the data (A-Ag^+^-T is also calculated to be non-planar, but given the small size of the T base there may still be A stacking). In the case of the mixed C_11_-G_11_ (Fig. 2), the predicted BE of G-Ag^+^-C (130.95 kcal/mol) is similar to G-Ag^+^-G (129.72 kcal/mol) and C-Ag^+^-C (126.18 kcal/mol) but the heterobase complexes are not detectable in the data for mixed G_11_ and C_11_ strands. The differences in hydration and stacking free energies may be what is dictating the preferential formation of homobase complexes. We also note that previous experimental work has detected C-Ag^+^-G pairings^31^, but only within hydrogen-bonded triplexes stabilized by multiple inter-strand T-A-T triplet pairings, with facing C bases embedded in the T-rich regions and facing G bases in A-rich regions. The homobase oligomers studied here provide an entirely different context.

In conclusion, our experimental studies of homo-base strands of DNA have identified an unanticipated Ag^+^-mediated pairing of guanine bases in homobase oligonucleotides. This discovery of the highly stable, silver-mediated pairing of G bases expands the diversity of known nontoxic metal-mediated interactions with natural DNA bases. Our complementary calculations do not constrain Ag^+^ to attach at base sites that correspond to WC pairing. Instead the unrestricted binding configurations identify that the most stable Ag^+^ attachment in G-Ag^+^-G pairs is to base sites that do not engage in WC pairing. Our results suggest that in mixed base, double stranded DNA with long enough runs of consecutive C or G bases, addition of sufficient Ag^+^ should reconfigure WC pairing to more stable Ag^+^-mediated base pairing. This expansion of the known interactions between silver cations and DNA bases paves the way for more robust DNA nanotechnology and for potential applications in biomedical science.

## Methods

### DNA preparation

DNA oligonucleotides were synthesized by Integrated DNA Technologies with standard desalting. We solvent-exchanged the strands to remove residual salts. All solutions used RNase/DNase-free distilled water (Life Technologies). AgNO_3_ was analytical grade (Sigma-Aldrich). Before each analysis, strands with and without Ag^+^ were annealed to 90 ^°^C for 5 minutes and allowed to cool slowly to ambient temperature.

### Mass spectrometry experiments

Samples for ESI-MS used 80 μM single-stranded DNA concentrations in 10 mM ammonium acetate buffer. Solutions of DNA with Ag^+^ contained a ratio of 0.5, 0.75 or 1.0 AgNO_3_ per base, as detailed in the main text. The oligonucleotide solutions were injected into the MS (Waters QTOF2) at 10 μL/min in ESI negative mode with a 2 kV capillary voltage, 30 V cone voltage and 10 V collision energy. The signal was integrated over approximately 5 minutes.

### Circular dichroism experiments

Samples for CD experiments used 17 μM single-stranded DNA concentrations in 7.5 mM MOPS buffer (pH = 7.0) containing approximately 2.5 mM Na^+^. All measurements were collected on an Aviv 202 circular dichrometer. For the measurements at 90 ^°^C, the samples were heated at a rate of 3 ^°^C/min and allowed 10 minutes to equilibrate at 90 °C before taking the full spectra. Blanks containing the appropriate concentration of buffer were collected before and after the samples, averaged and then subtracted from the sample spectra to correct for background signal.

## Author Contributions

S.M.S. and E.G.G. conceived and designed the experiments. L.E.L. and O.L.A. conceived and designed the calculations, with initial suggestions from E.G.G. S.M.S. carried out the experiments and J.P. provided additional expertise in ESI-MS. S.M.S. and E.G.G. analyzed and interpreted data and wrote the experimental parts of the manuscript. L.E.L. and O.L.A. analyzed the computational information and wrote the theoretical parts of the manuscript. All authors reviewed the manuscript.

## Additional Information

**How to cite this article**: Swasey, S. M. *et al.* Silver (I) as DNA glue: Ag^+^-mediated guanine pairing revealed by removing Watson-Crick constraints. *Sci. Rep.* **5**, 10163; doi: 10.1038/srep10163 (2015).

## Supplementary Material

Supplementary Information
