# Supplementary material for: Silver (I) as DNA glue: Ag+-mediated guanine pairing revealed by removing Watson-Crick constraints
Source: Sci Rep. 2015 May 14;5:10163. doi: 10.1038/srep10163 (PMC4431418; doi:10.1038/srep10163)
Supplement: Supplementary Information [file srep10163-s1.pdf]

Supplementary information

Silver (I) as DNA glue: Ag^+-mediated guanine pairing revealed by removing Watson-Crick constraints

Steven M. Swasey[b], Leonardo Espinosa Leal[c], Olga Lopez-Acevedo[c], James Pavlovich[b], Elisabeth G. Gwinn*[a]

*[a] Prof. E. G. Gwinn
Department of Physics
UCSB
Santa Barbara, CA 93117
E-mail: bgwinn@physics.ucsb.edu

[b] S. M. Swasey, J. Pavlovich
Department of Chemistry and Biochemistry
UCSB

[c] Dr. O. Lopez-Acevedo, Dr. L. E. Leal
Department of Applied Physics
Aalto University
00076 Aalto, Finland

Supporting Information Index

Mass spectra p. 2-7

M/Z for high abundance products p. 8

Computational Details p. 9-13

Computed Structures p. 14-20

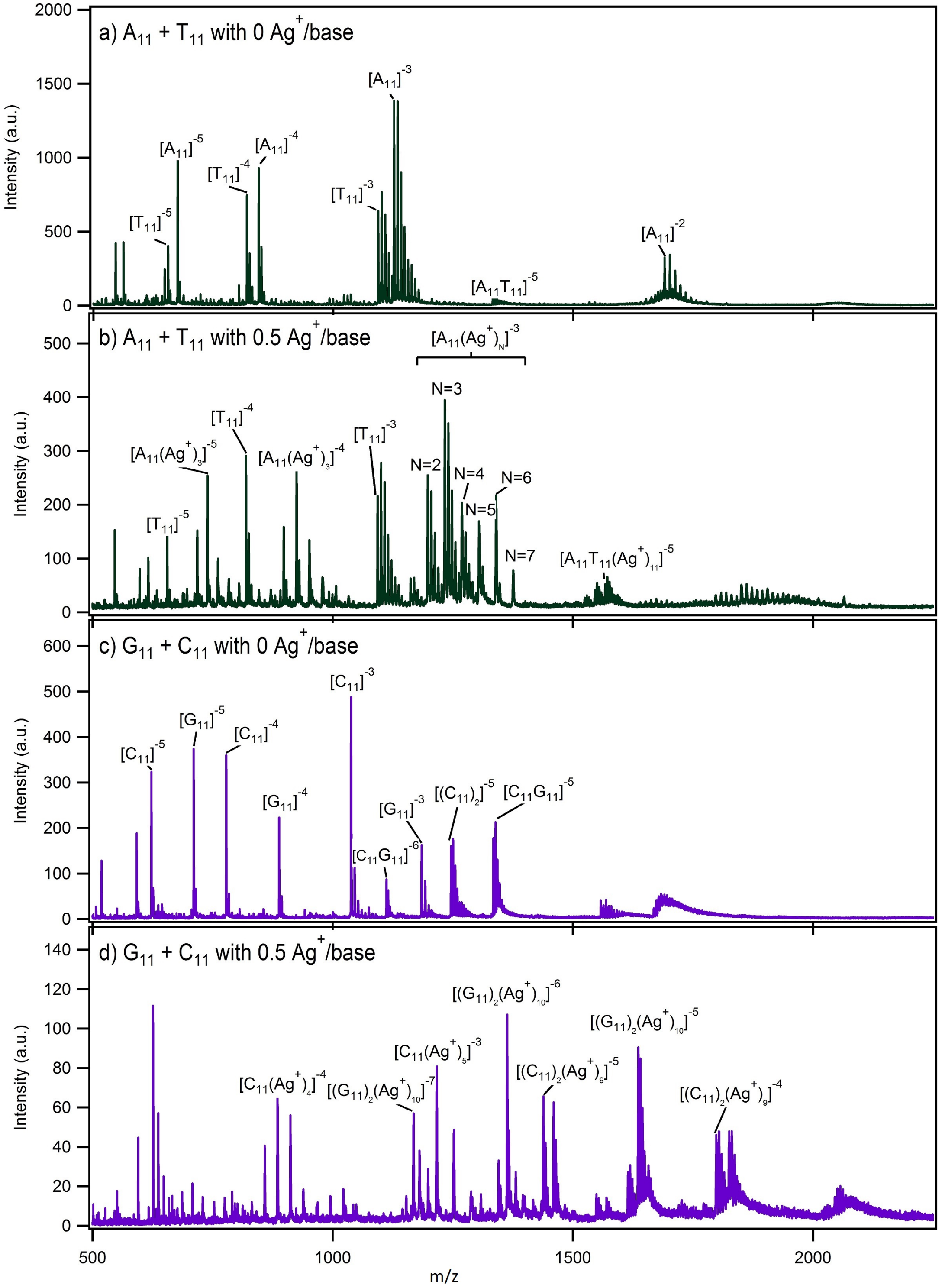

Figure S1) Full range mass spectra of solutions containing A_{11} and T_{11} with a) no Ag^+ and b) $0.5Ag^+$/base as well as G_{11} and C_{11} with c) no Ag^+ and d) 0.5 Ag^+/base. The products are labeled in the format $[(X_{11})_y(Ag^+)_N]^Z$ where X is the base of the homobase strand, y is the number of homobase strands in the product, N is the number of cationic silver atoms, and z is the charge state. z is negative, and is given by $z = N_{Ag}^+ - n_{pr}$, where n_{pr} is the number of protons removed from the DNA, and N_{Ag+} is the number of attached silver cations.

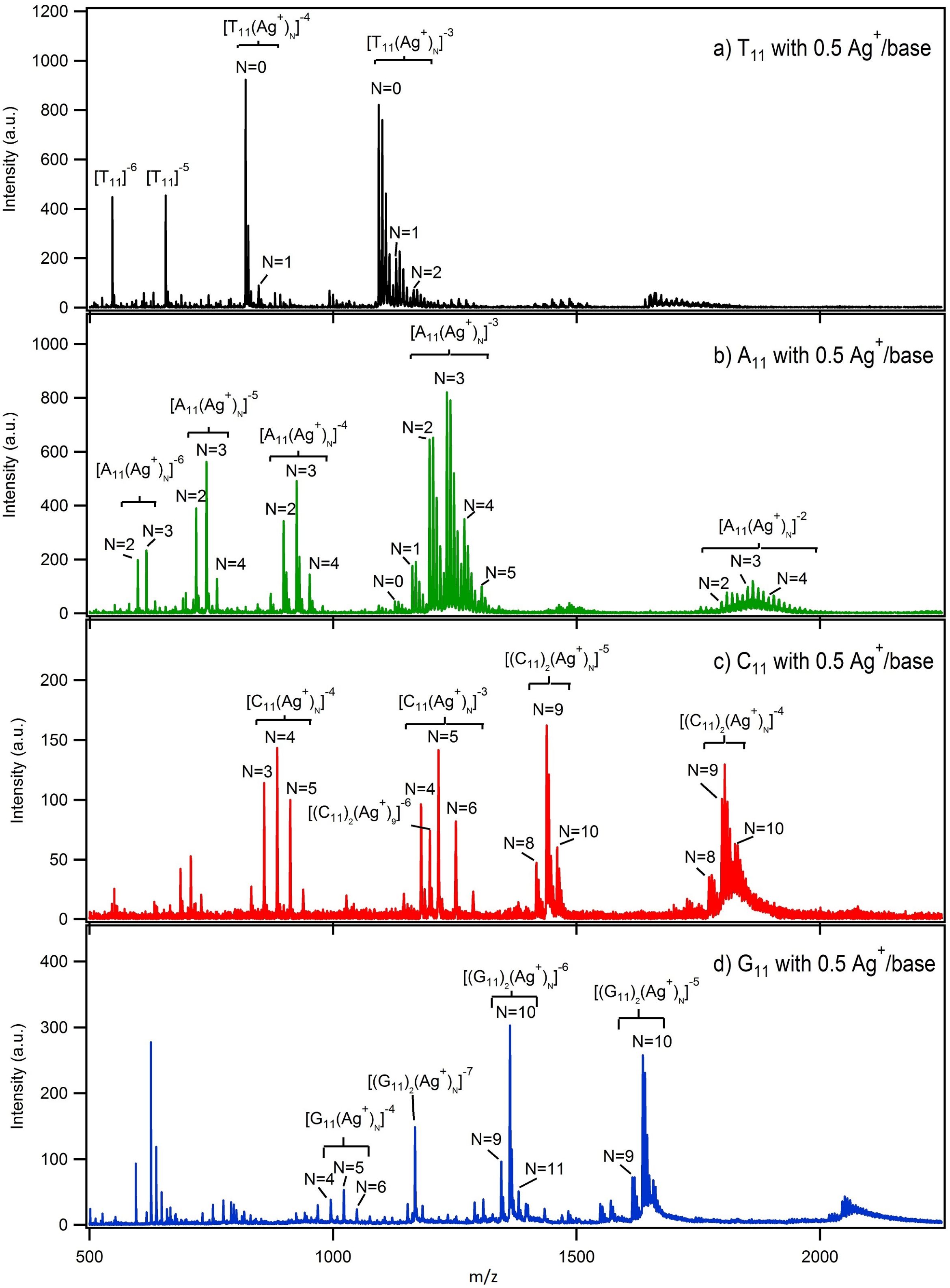

Figure S2) Full range mass spectra of a) T_{11} b) A_{11} c) C_{11} and d) G_{11} at 80μM DNA concentration and 0.5 Ag^{+}/base ratio taken in ESI negative mode.

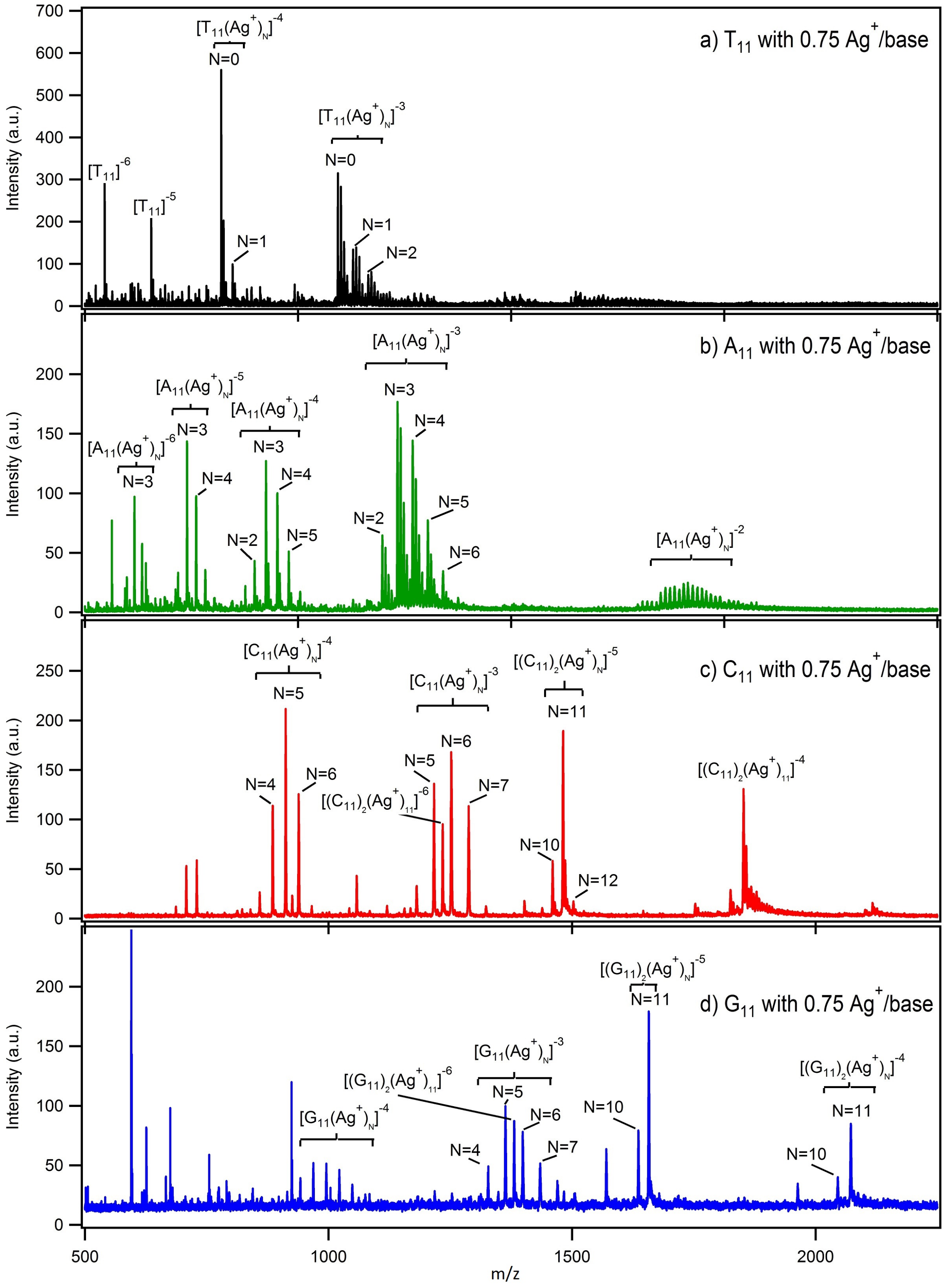

Figure S3) Full range mass spectra of a) T_{11} b) A_{11} c) C_{11} and d) G_{11} at 80μM DNA concentration and 0.75 Ag^{+}/base ratio taken in ESI negative mode.

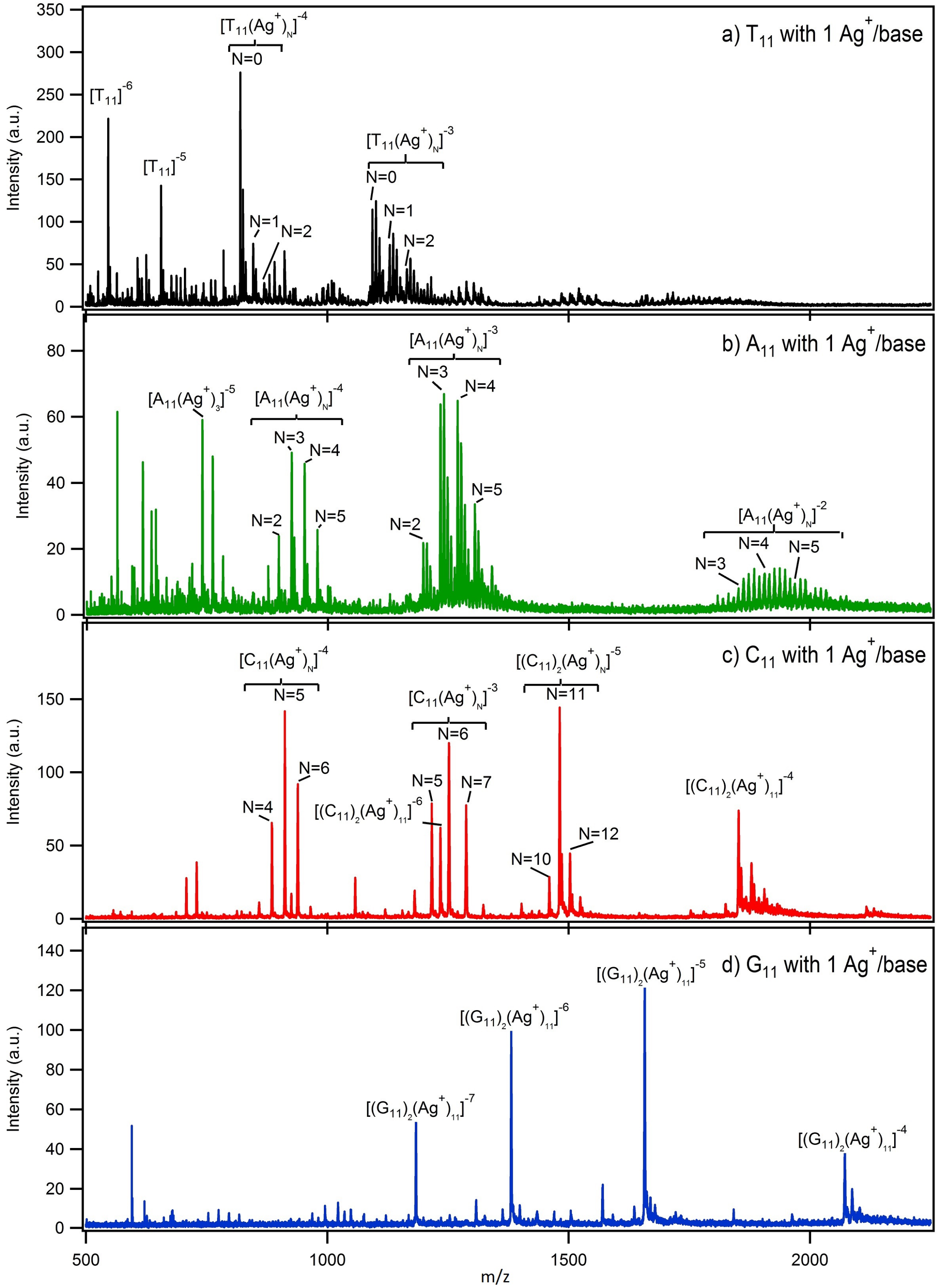

Figure S4) Full range mass spectra of a) T_{11} b) A_{11} c) C_{11} and d) G_{11} at 80µM DNA concentration and 1 Ag^{+}/base ratio taken in ESI negative mode.

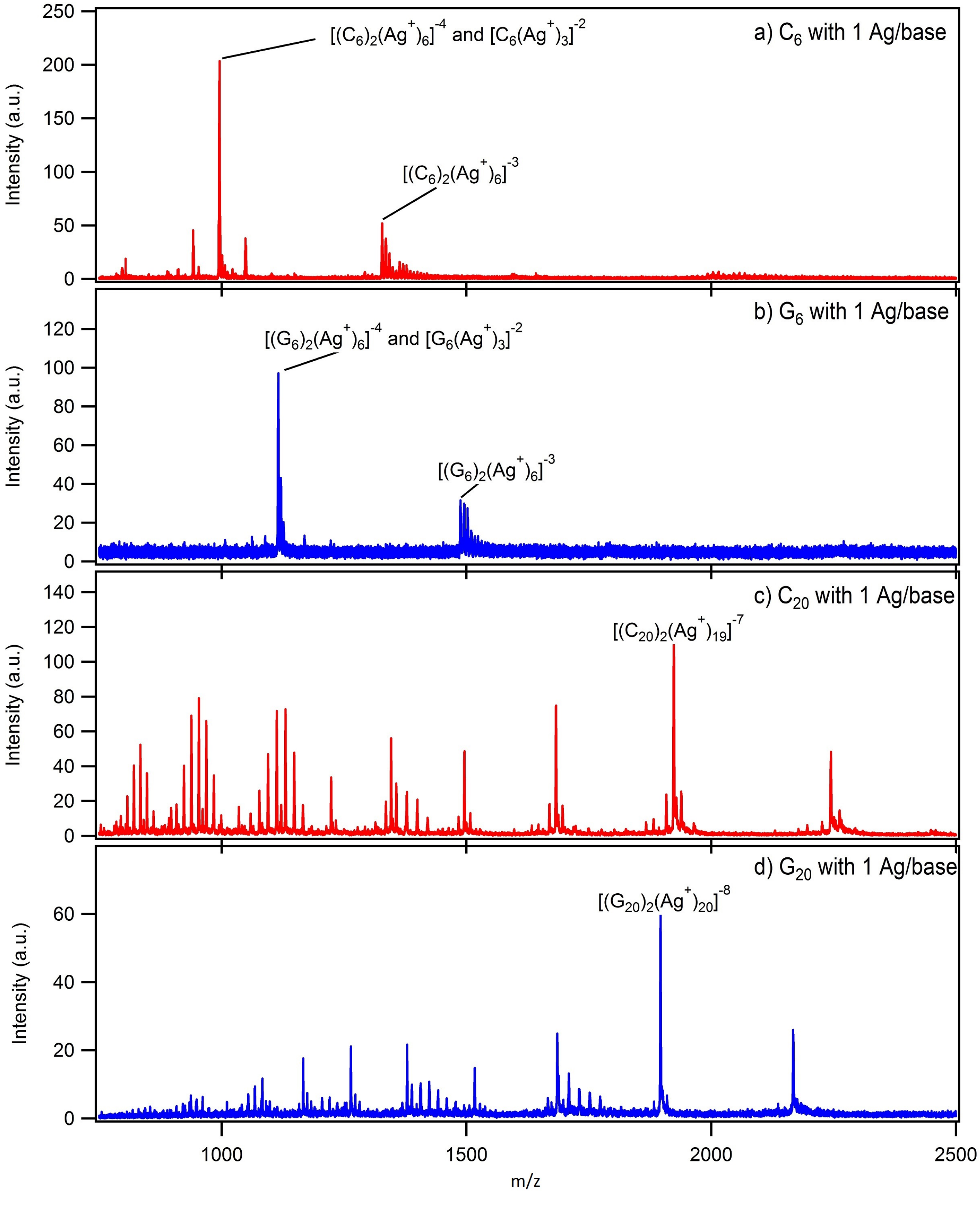

Figure S5) Mass spectra of a) C_6, b)G_6, c) C_{20} and d) G_{20} at 80µM DNA concentration and 1 Ag^+/base ratio taken in ESI negative mode. Certain peaks have multiple assignments due to overlapping charge states of dimer and monomer products.

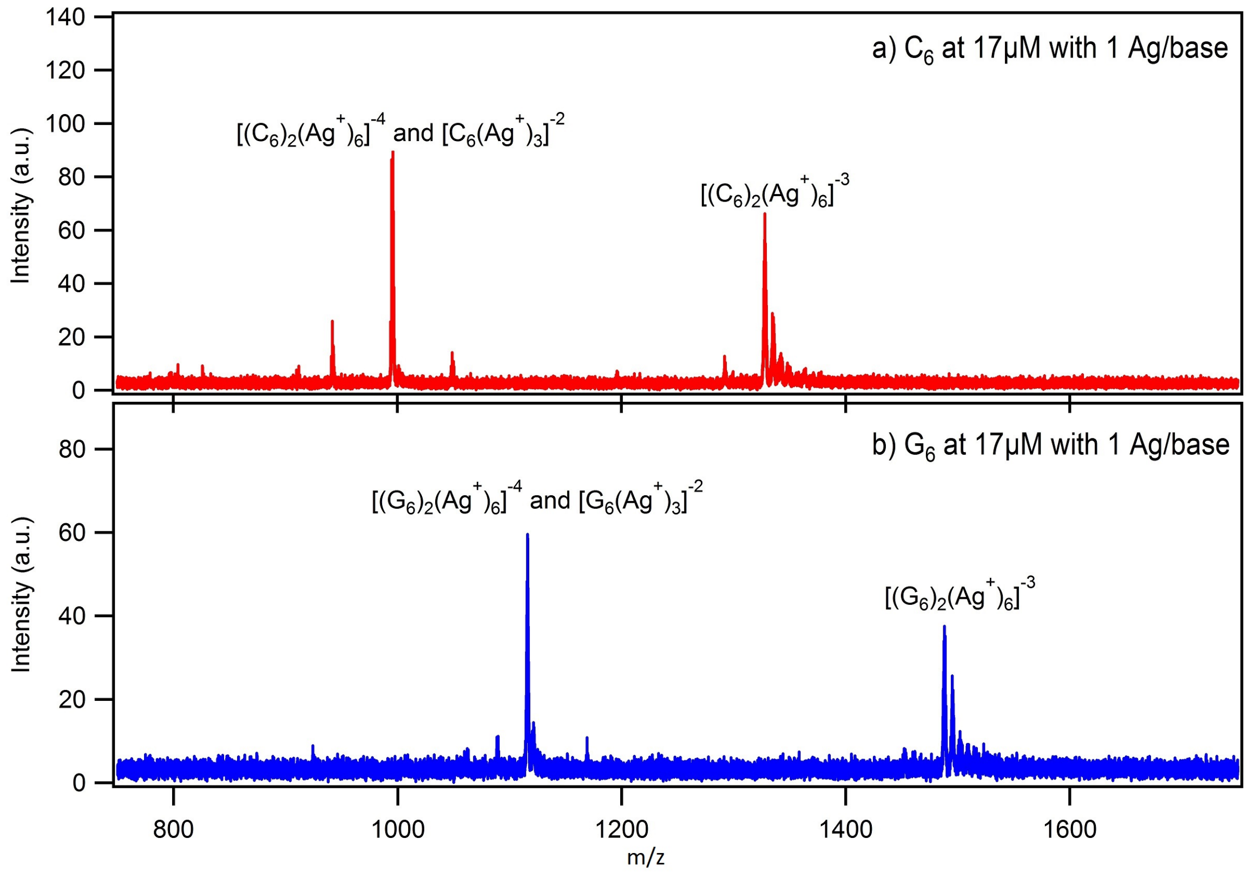

Figure S6) Mass spectra of a) C_6 and b) G_6 at strand concentrations used for circular dichroism experiments (17 μM) and 1 Ag^+/base ratio taken in ESI negative mode. Certain peaks have multiple assignments due to overlapping charge states of dimer and monomer products.

Table S1) m/z values for highly abundant charge state products obtained from the mass spectra in Figures S1-S2 and the corresponding numbers of attached silver cations, N, rounded to an integer from the experimental value in parentheses. The small difference is due to experimental uncertainty in the calibration of the m/z axis.

Product	N	m/z
$[T_{11}(Ag^+)_N]^{-4}$	0	819.89
	1 (0.99)	846.60
	2 (1.99)	873.32
$[A_{11}(Ag^+)_N]^{-3}$	0	1126.60
	1 (1.00)	1162.15
	2 (2.00)	1197.80
	3 (3.00)	1233.43
	4 (4.00)	1269.07
	5 (5.00)	1304.70
	6 (6.00)	1340.33
	7 (7.01)	1376.31
$[A_{11}T_{11}(Ag^+)_N]^{-5}$	0	1332.48
	9 (9.00)	1524.91
	10 (10.01)	1546.44
	11 (11.00)	1567.68
	12 (12.00)	1589.00
$[(C_{11})_2(Ag^+)_N]^{-5}$	9 (9.00)	1438.93
	10 (10.00)	1460.34
	11 (10.99)	1481.53
	12 (12.00)	1503.11
	13 (12.99)	1524.31

Product	N	m/z
$[C_{11}]^{-3}$	N/A	1038.54
$[C_{11}(Ag^+)_N]^{-4}$	3 (2.99)	858.77
	4 (4.00)	885.50
	5 (5.00)	912.23
	6 (6.01)	939.20
	7 (6.99)	965.64
$[(C_{11})_2]^{-5}$	N/A	1246.60
$[G_{11}(Ag^+)_N]^{-4}$	2 (2.00)	942.30
	3 (3.00)	969.03
	4 (3.99)	995.51
	5 (4.99)	1022.23
	6 (6.00)	1049.18
	7 (7.00)	1075.66
	8 (8.00)	1102.63
$[G_{11}]^{-5}$	N/A	710.71
$[(G_{11})_2(Ag^+)_N]^{-5}$	10 (10.00)	1636.34
	11 (11.00)	1657.75
	12 (12.00)	1679.34
$[C_{11}G_{11}]^{-5}$	N/A	1334.61

The masses of A_{11}, C_{11}, G_{11} and T_{11} are 3383.3, 3119.1, 3559.3, and 3284.2 g/mol respectively. We use the separately determined ion mass, m, and charge state, z, from the isotope peak envelope (as exemplified in the main text, Fig. 1b) to obtain the silver cation composition. The measured charge state, z, depends on the difference in the number of protons removed from the DNA and the number of attached Ag^+.

The number of silver cations, N, is given by $N = [m-(X_{11}+zm_p)] /(m_{Ag}-m_p)$ where X_{11} is the molecular weight of the (unionized) homobase strand (or the mass of two strands, in the case of a dimer product); m_{Ag} = 107.868 g/mol is the atomic weight of silver and m_p = 1.007 g/mol is the mass of a proton. This calculation assumes all silver is cationic, verified through calculated isotope peak fitting to the high resolution mass spectra as described in the main text. (The presence of any neutral silver atoms would shift the isotope peak pattern by reducing the number of protons that must be removed to reach the measured charge state, z).

Computational results

A high number of incorporated silver ions per duplex means that we can expect high symmetry. Indeed, the model proposed experimentally for a silver mediated cytosine duplex contains repeated C-Ag^+-C units, each in a trans configuration. The trans C-Ag^+-C is also the ground-state structure we obtained at DFT level in vacuum [52]. In those simulations, including an implicit solvent decreased the binding energy but did change the relative ordering of the binding energies of the various base-Ag^+ and base-Ag^+-base complexes considered. In this computational section, we consider as a model of the metal mediated duplex the trans C-Ag^+-C that would be repeated along the backbone axis. This trimer is comprised of a base homodimer and a silver atom in vacuum, and the overall charge is set to +1. Starting from various initial conditions, comprising WC-type and not WC-type, we first apply a global optimization followed by a final force optimization.

The binding strengths from this the metal mediated dimer model agree with quantitative results obtained in the mass spectroscopy experiment. From calculation of binding energies, the ordering G ~ C > A > T is obtained. Respectively in kcal/mol the binding energies, defined as sum of energy of fragments minus the energy of the complex, are 129.72 ~ 126.17 > 111.90 > 91.53 for the PBE-TS09 calculations, which used a real-space basis and the projector-augmented wave method (PAW). The values obtained with the real-space PAW method does not give inaccuracies due to basis set superposition errors (BSSE) (See explanation below). The same ordering with binding energies of 116.15 ~ 115.26 > 94.78 > 78.28 kcal/mol is obtained with CAM-B3LYP/6-311+G(d,p), an average shift of 13.7 kcal/mol but with a much smaller standard deviation of the shifts, 2.6 kcal/mol, reflecting the general feature that DFT methods give good *relative* energies. Here the BSSE energy subtracted to find the total binding energy is 1.27, 1.54, 1.29 and 1.07 kcal/mol, respectively. Using instead the Minnesota functional M06-L, the results are 112.57 ~ 112.22 > 91.80 > 74.54 kcal/mol, with BSSE energy correction of 1.29, 1.19, 1.03 and 0.98 kcal/mol. This is in excellent agreement with our other Gaussian basis calculations (CAM-B3LYP), with an average shift of 3.3 kcal/mol and a quite small standard deviation of the shift, 0.4 kcal/mol. With the M06-L there is a switch in the order of the binding energies for C-Ag^+-C and G-Ag^+-G, from G~C>A>T to C ~ G > A > T. However, the values are very close to each other.

As the measure of co-planarity of the bases in the trimer complexes, we use a dihedral angle. C-Ag^+-C and G-Ag^+-G are nearly planar while T-Ag^+-T and A-Ag^+-A are significantly non-planar. This agrees with the experimental findings of a metal mediated duplex of G DNA oligomers and C DNA oligomers, but no metal mediated duplex of T DNA oligomers or A DNA oligomers. The G-Ag^+-G ground state is non WC type and the silver binds through the N7 atom in each base. In contrast, the C-Ag^+-C ground state is WC type and the silver bridges the N3 atom of each base. In both cases a hydrogen bond helps stabilize the complexes. T-Ag^+-T binding energy is much lower and the ground state is non planar with no local minima close to planar configuration. This qualitatively accounts for the experimental results that at most 2 silver cations to attach to T_{11} DNA strands, with the bare T_{11} strand the dominant product even at 1 Ag^+ added per base (Fig. 1a,b,c). In contrast to the other bases, A-Ag^+-A has many configurations close in energy, with 14 in an energy interval of 8 kcal/mol (0.35 eV). The many equivalent binding sites of

adenine may explain the higher numbers of silver cations (up to 6) attached to individual A_{11} DNA strands in the data (Fig. 3 d-f).

For completeness, we also explored CG and AT dimers mediated by silver cations. The C-Ag^+-G complex's ground state is non-WC type and planar. This bonding configuration has been reported earlier in silver mediated GCG triplexes [31, 36]. The metal-AT dimer's ground state is non WC-type and non-planar.

Planarity of metal mediated homodimers:
The dihedral angles (see Fig. S3 for the labeling in all bases) are C8-N7-N7-C8 = 181.2º (180º with CAM-B3LYP) for G-Ag^+-G and C2-N3-N3-C3 = 172º (180º with CAM-B3LYP) for C-Ag^+-C. A quite different configuration is obtained for A-Ag^+-A and T-Ag^+-T where the ground state is non-planar. For A-Ag^+-A, C2-N3-N3-C2 has an angle of 101.6º (84.70º for C2-N1-N1-C2 with CAM-B3LYP). The binding site of A-Ag+-A depends then on the functional used. This is the only ground state where PBE+TS09 and CAM-B3LYP disagree. With PBE+TS09 functional it was found that there is a large rotational freedom for the ground state for the A-Ag^+-A dimer. The difference in energy for the perpendicular dimer and a dimer in a local minimum close to planar is around 0.7 kcal/mol (0.03 eV). For T-Ag^+-T, the C2-N3-N3-C2 dihedral angle was -140º degrees (-129.26º with CAM-B3LYP).

Metal bound to single bases:
In the lowest energy Ag^+-G structure, the silver binds to N7-O atoms in the G base with binding energy of 77.08 kcal/mol (70.39 with CAM-B3LYP). In the Ag^+-C case, the silver binds to the central nitrogen with 71.16 kcal/mol of binding energy (64.73 using CAM-B3LYP).

Metal mediated GC and AT dimers:
The most stable non-WC G-Ag^+-C geometry has a binding energy of 130.95 kcal/mol (120.16 with CAM-B3LYP) and a dihedral angle, defined by G[C2]-C[N3]-G[N7]-G[C5], of 183º (180º with CAM-B3LYP).The structure has earlier been studied computationally (Ref 28 in main text) and has been observed as part of a triplex (Ref 23 in main text). Non-WC A-Ag^+-T has a binding energy of 102.08 kcal/mol (87.63 with CAM-B3LYP) and the dihedral angle for T[C4]-T[O4]-A[N3]-A[C4] is 129.8º (126.3º with CAM-B3LYP). For the WC G-Ag^+-C geometry the binding energy is 116.64 kcal/mol (104.96 with CAM-B3LYP) and the dihedral angle of C[C2]-C[N3]-G[O6]-G[C6] is 3.7º (0.0 º with CAM-B3LYP). For the WC A-Ag^+-T geometry the binding energy is 101.22 kcal/mol (87.81 with CAM-B3LYP) and the angle defined by T[C4]-T[O4]-A[N1]-A[C6] is 155.6º (125.7º with CAM-B3LYP). For the deprotonated (denoted with a *) silver mediated WC base pairs the binding energy is defined differently to include the change in electrons and protons from final structure and components. The binding energy is computed as the difference between the system composed of a trimer and a water ion and the energy of the separated bases, silver ion and water molecule: $E(T^*\text{-}Ag^+\text{-}A)+E(H_3O^+)-E(T)-E(A)-E(Ag^+)-E(H_2O)$. An equivalent definition is used for G*C. For T*-Ag^+-A the binding energy is 30.01 kcal/mol (15.72 with CAM-B3LYP) and for G*-Ag^+-C is 37.07 kcal/mol (24.62 with CAM-B3LYP). Both T*-Ag^+-A and G*-Ag^+-C are fully planar.

Table S2) Summary of all structures including: structure label (str), binding energies obtained with PBE+vdW, dihedral angles and atom-metal-atom bond lengths and angles. The notation “WC” means that Ag^+ binds at base sites for Watson-Crick pairing. The **boldface entries** are for the most stable structure. For reference the values of the binding energies obtained with the Gaussian code are in

parentheses. The values marked with asterisk (*) in the fourth column corresponds to structures where the Ag^+ binds to only one nucleobase in the pair. The labeling of the figures on the following pages follows the labeling convention in this table.

<u>A-Ag^+-A (see Fig. S8)</u>

str	Binding (kcal/mol)	dihedral angle (degrees)	bond length (Å) (angle -degrees)
WC			
1	109.60	C2-N1-N1-C2: 342.4	2.108-2.109 (174.6)
2	110.10	C2-N1-N1-C2: 179.1	2.097-2.097 (176.6)
3	110.96	C2-N1-N1-C2: 265.0	2.107-2.104 (178.3)
**4**	**111.90 (96.07)**	**C2-N3-N3-C2: 101.6**	**2.087-2.087 (178.8)**
non-WC			
1	110.51	C2-N1-N3-C2: 174.3	2.099- 2.088 (178.5)
2	109.45	C2-N1-N3-C2: 353.2	2.108-2.101 (173.8)
3	106.94	C2-N1-N7-C9: 178.5	2.097-2.083 (177.3)
4	106.80	C2-N1-N7-C9: 9.0	2.104-2.093 (171.0)
5	109.51	C9-N7-N7-C9: 357.5	2.098-2.094 (168.7)
6	111.23	C2-N3-N3-C2: 175.6	2.093-2.093 (178.4)
7	106.44	C8-N7-N3-C2: 359.5	2.085-2.090 (177.7)
8	107.52	C8-N7-N3-C2: 174.8	2.089-2.093 (177.8)
9	106.12	C8-N7-N7-C8: 14.2	2.081-2.080 (177.2)
10	103.88	C8-N7-N7-C8: 180.6	2.086-2.086 (176.5)

<u>C-Ag^+-C (see Fig. S9)</u>

str	Binding (kcal/mol)	dihedral angle (degrees)	bond length (Å) (angle -degrees)
WC			
1	121.40	C2-N3-N3-C2: 311.1	2.124-2.123 (178.4)
**2**	**126.18 (116.08)**	**C2-N3-N3-C2: 171.9**	**2.104-2.114 (169.2)**
non-WC			
1	121.19	C2-O2-N3-C2: 3.3	2.133-2.121 (170.7)
2	119.29	C2-O2-N3-C2: 157.6	2.100-2.108 (177.9)
3	117.15	C2-O2-O2-C2: 173.3	2.124-2.133 (179.4)
4	95.23	O2-C2-O2-C2: 176.4	2.228-2.492 (57.4)
5	97.49	O2-C2-O2-C2: 8.1	2.241-2.439 (58.0)

<u>G-Ag^+-G (see Fig. S10)</u>

str	Binding (kcal/mol)	dihedral angle (degrees)	bond length Å) (angle -degrees)
WC			
1	104.12	O6-C6-C6-O6: 158	2.369-2.247 (81.6)
2	102.67	C8-N7-N6-C6: 192.5	2.107- 2.171 (172.5)
3	97.74	C6-O6-O6-C6: 17.5	2.069- 2.070 (177.4)
4	71.50	C2-N2-N2-C2: 340.7	2.162- 2.167 (173.6)

non-WC			
1	110.52	C6-O6-N3-C4: 176.3	2.099- 2.084 (179.2)
2	110.69	C6-O6-N3-C4: 358.7	2.089- 2.081 (177.6)
3	125.32	C5-N7-N7-C5: 22.5	2.105- 2.107 (166.2)
4	128.57	C8-N7-N7-C8: 186.8	2.100- 2.091 (166.2)
5	118.67	C8-N7-O6-C6: 159.6	2.106- 2.125 (157.8)
6	129.56	C8-N7-N7-C8: 179.0	2.091- 2.097 (175.0)
**7**	**129.72 (117.77)**	**C8-N7-N7-C8: 181.2**	**2.094- 2.094 (176.9)**
8	108.53	C6-O6-O6-C6: 358.9	2.082- 2.111 (166.8)

T-Ag$^+$-T (see Fig. S11)

str	Binding (kcal/mol)	dihedral angle (degrees)	bond length (Å) (angle -degrees)
WC			
1	90.88	C4-O4-O4-C4: 351.1	2.086-2.086 (174.4)
2	88.58	C4-O4-O2-C2: 357.3	2.078-2.086 (173.0)
3	85.45	C2-O2-O2-C2: 348.1	2.088-2.088 (179.6)
**4**	**91.53 (79.35)**	**C4-O4-O4-C4: -140.0**	**2.088-2.087 (173.9)**

A-Ag$^+$-T (see Fig. S12)

str	Binding (kcal/mol)	dihedral angle (degrees)	bond length (Å) (angle -degrees)
WC			
1	76.30	A[C2]-A[N1]-T[N3]-T[C2]: 177	2.113 A(N7)*
2	91.39	A[C6]-A[N6]-T[O4]-T[C4]: 339.7	2.107-2.159 (165.7)
**3**	**101.22 (87.81)**	**A[C6]-A[N1]-T[O4]-T[C4]: 155.6**	**2.093-2.085 (179)**
4	98.57	A[C2]-A[N1]-T[O2]-T[C2]: 125.7	2.099-2.100 (178.6)
5	73.90	A[C2]-A[N1]-T[N3]-T[C2]: 349.2	2.136- 2.804 T(73.1)*
6	80.65	A[C2]-A[N1]-T[N3]-T[C2]: 359.3	2.122 A(N3)*
7	98.80	A[C2]-A[N1]-T[O2]-T[C2]: 67.1	2.095-2.090 (174.5)
non-WC			
1	98.90	A[C2]-A[N1]-T[O2]-T[C2]: 358.8	2.092- 2.093 (178.4)
2	99.00	A[C2]-A[N3]-T[O2]-T[C2]: 337.5	2.082- 2.090 (171.4)
3	97.35	A[C2]-A[N3]-T[O2]-T[C2]: 155.6	2.094-2.094 (164.8)
4	95.55	A[C8]-A[N7]-T[O2]-T[C2]: 350.4	2.077- 2.089 (167.3)
5	95.09	A[C8]-A[N7]-T[O2]-T[C2]: 170.6	2.083 -2.087 (169.9)
6	95.84	A[C8]-A[N7]-T[O4]-T[C4]: 241.7	2.074 – 2.066 (175.1)
7	98.61	A[C8]-A[N7]-T[O4]-T[C4]: 31.3	2.075-2.083 (166.1)
8	101.32	A[C2]-A[N3]-T[O4]-T[C4]: 355.4	2.084- 2.087 (169.2)
9	98.86	A[C2]-A[N3]-T[O4]-T[C4]: 19.0	2.071- 2.082 (178.4)
10	98.57	A[C2]-A[N3]-T[O4]-T[C4]: 116.8	2.071- 2.091 (179.5)
11	98.29	A[C2]-A[N1]-T[O4]-T[C4]: 251.1	2.080 – 2.099 (167.5)
**12**	**102.08 (87.63)**	**A[C4]-A[N3]-T[O4]-T[C4]: 129.8**	**2.076- 2.074 (173.5)**

<u>G-Ag$^+$-C (see Fig. S13)</u>

str	Binding (kcal/mol)	dihedral angle (degrees)	bond length (Å) (angle -degrees)
WC			
1	112.82	G[C2]-G[N1]-C[N3]-C[C2]: 177.3	2.431- 2.211 G(80.6)*
**2**	**116.64 (104.96)**	**G[C6]-G[O6]-C[N3]-C[C2]: 3.7**	**2.118- 2.086 (163.2)**
3	106.59	G[C2]-G[N2]-C[O2]-C[C2]: 12.9	2.149- 2.083 (170.5)
4	81.13	G[C2]-G[N1]-C[N3]-C[C2]: 352.8	2.150 C(O2)*
5	92.02	G[C2]-G[N1]-C[N3]-C[C2]: 352.7	2.115 G(N3)*
non-WC			
1	130.67	G[C8]-G[N7]-C[N3]-C[C2]: 355.5	2.094-2.102 (170.5)
2	120.22	G[C6]-G[O6]-C[N3]-C[C2]: 192.0	2.107-2.105 (176.4)
3	111.89	G[C2]-G[N3]-C[N3]-C[C2]: 185.0	2.095-2.118 (168.0)
4	110.62	G[C2]-G[N3]-C[N3]-C[C2]: 3.2	2.092-2.112 (171.4)
5	114.84	G[C6]-G[O6]-C[N3]-C[C2]: 355.8	2.073-2.103 (178.3)
6	107.60	G[C6]-G[O6]-C[O2]-C[C2]: 358.1	2.091-2.102 (175.0)
7	106.25	G[C2]-G[N2]-C[O2]-C[C2]: 0.5	2.150-2.088 (172.3)
8	122.11	G[C6]-G[O6]-C[N3]-C[C2]: 171.5	2.116-2.100 (173.2)
**9**	**130.95 (120.16)**	**G[C8]-G[N7]-C[N3]-C[C2]: 358.8**	**2.092-2.102 (167.5)**
10	106.90	G[C2]-G[N3]-C[O2]-C[C2]: 9.7	2.095-2.093 (177.4)
11	106.60	G[C2]-G[N3]-C[O2]-C[C2]: 183.7	2.087- 2.089 (174.4)

<u>Deprotonated (see Fig. S14)</u>

str	Binding (kcal/mol)	dihedral angle (degrees)	bond length (Å) (angle -degrees)
T*-Ag$^+$-A	30.09 (15.72)	A[C2]-A[N1]-T[N3]-C[C2]:355.0	2.116-2.079 (160.3)
G*-Ag$^+$-C	37.07 (24.62)	G[C2]-G[N1]-C[N3]-C[C2]:353.0	2.097-2.121 (157.4)

The PAW method:

The computational results in the main text were calculated with a grid based code (GPAW) that does not present the problem of superposition errors of atomic basis. GPAW could present a similar type of superposition error if the bond distances between two atoms where smaller than the sum of cutoff radius where the PAW transformation is applied. Cutoff values are chosen small enough to avoid this problem, as shown in Table S3.

The dataset used in the stable release of GPAW uses a variable named rcut that determines the size of the sphere around each atom where the PAW transformation is applied. We are providing here the maximum rcut (there is one per valence atomic orbital) in the setups released on Mar 27 2014 version 0.9 revision 11271.

Table S3) Values of rcut (in Bohr) for the different atoms in the studied molecular structures of this work. The van der Waals radius is giving for comparison:

atom	rcut	vdW
H	0.9	2.27
C	1.2	3.21
O	1.3	2.87
N	1.14	2.93
Ag	2.51	3.25

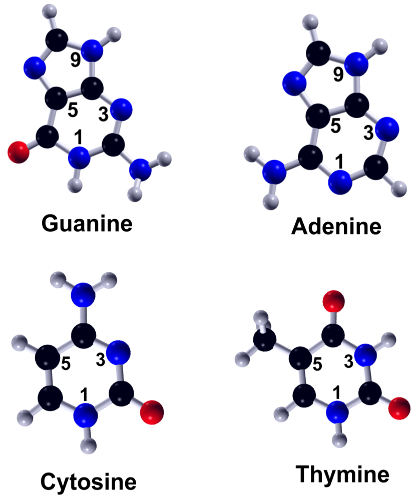

Figure S7) Labels used in this work for the definition of the dihedral angles in the four DNA bases

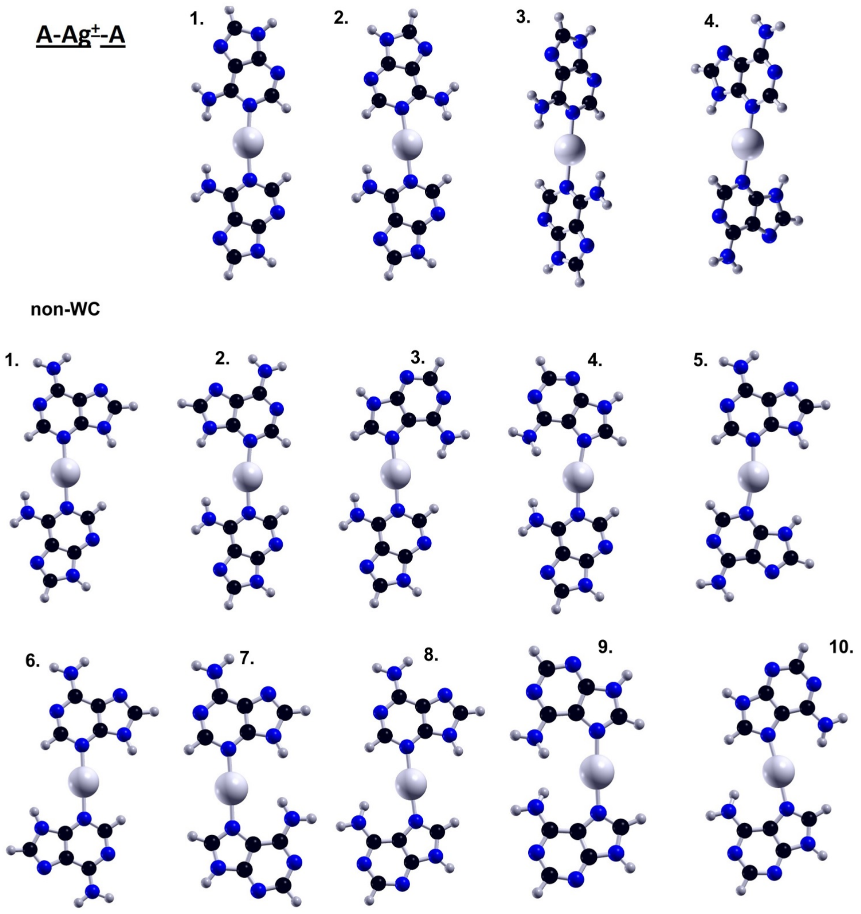

Figure S8) Representation of the optimized geometries for the A-Ag^+-A pairs (see Table S2) obtained by means of Density Functional theory (DFT) at PBE+vdW level.

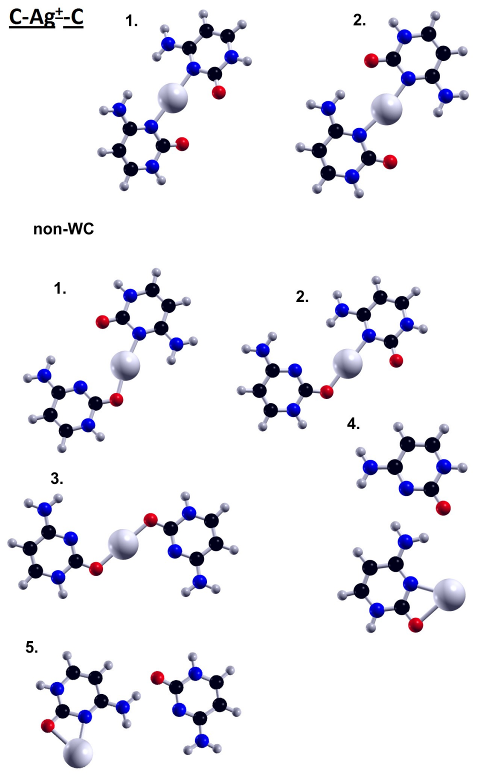

Figure S9) Representation of the optimized geometries for the C-Ag^{+}-C pairs (see Table S2) obtained by means of Density Functional theory (DFT) at PBE+vdW level.

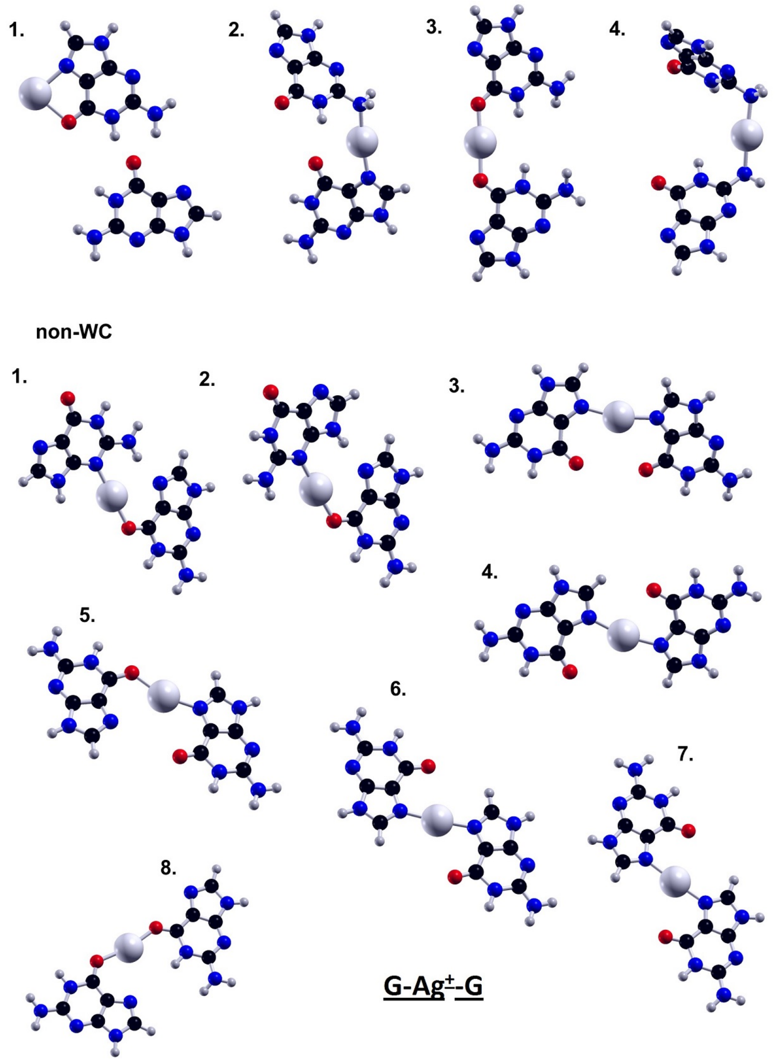

Figure S10) Representation of the optimized geometries for the G-Ag^+-G pairs (see Table S2) obtained by means of Density Functional theory (DFT) at PBE+vdW level.

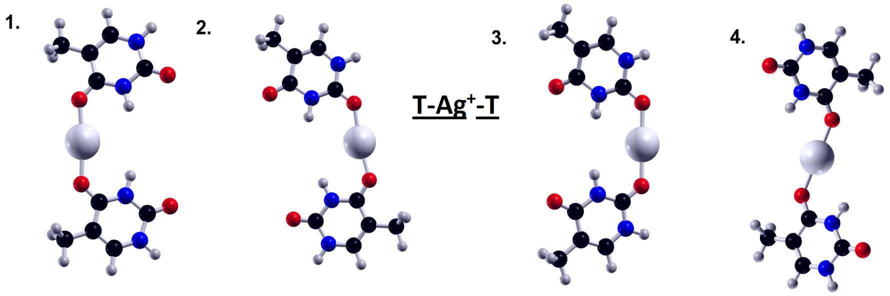

Figure S11) Representation of the optimized geometries for the T-Ag^+-T pairs (see Table S2) obtained by means of Density Functional theory (DFT) at PBE+vdW level.

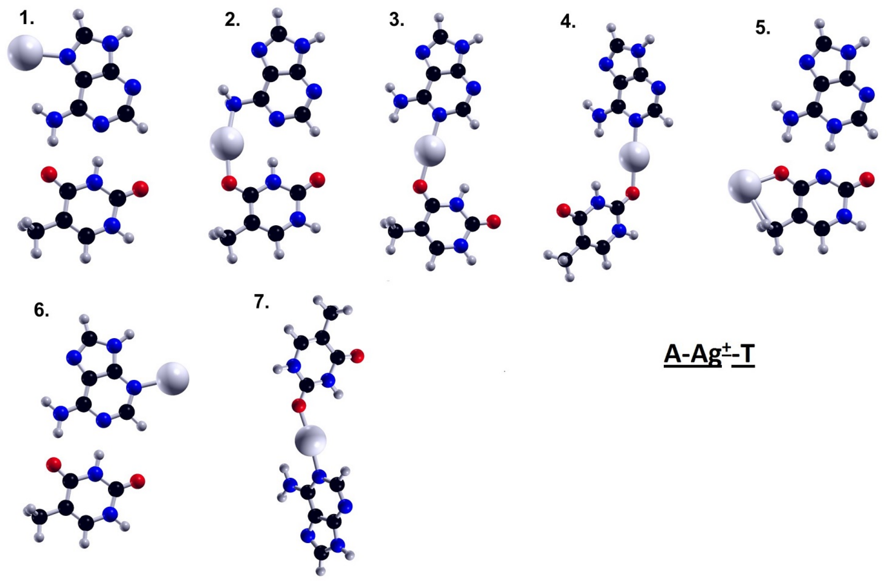

non-WC

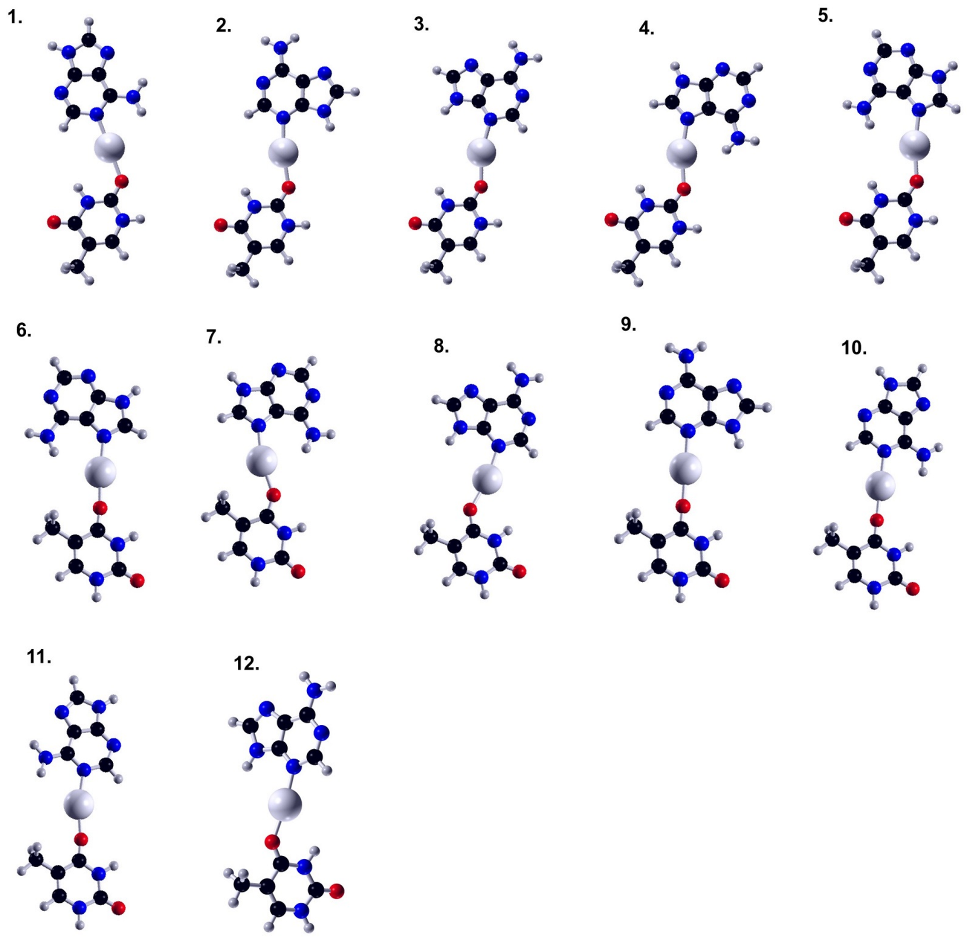

Figure S12) Representation of the optimized geometries for the A-Ag$^+$-T pairs (see Table S2) obtained by means of Density Functional theory (DFT) at PBE+vdW level.

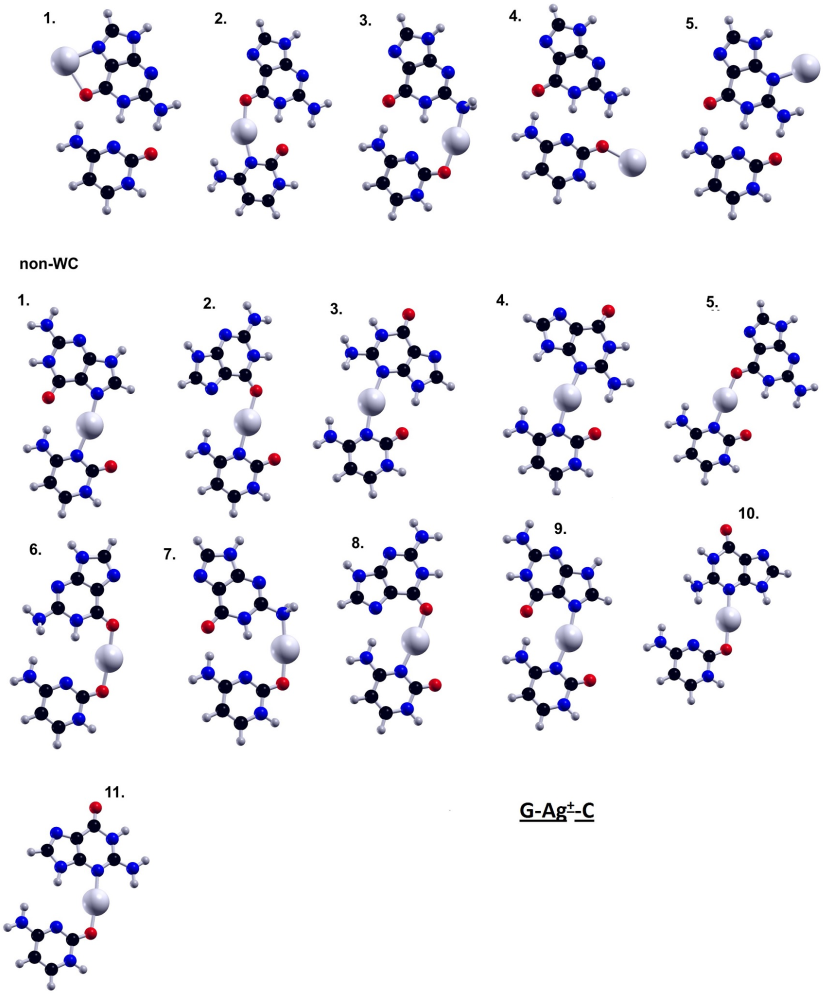

Figure S13) Representation of the optimized geometries for the G-Ag^{+}-C pairs (see Table S2) obtained by means of Density Functional theory (DFT) at PBE+vdW level.

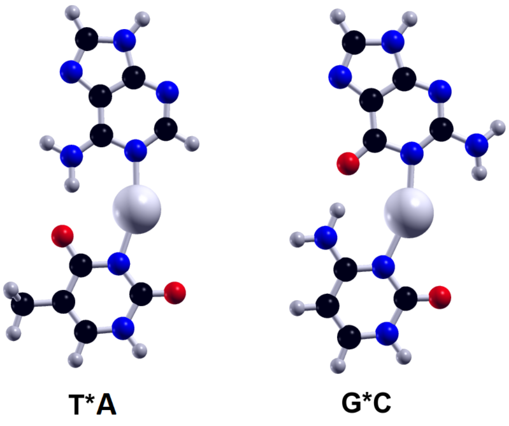

Figure S14) Representation of the optimized geometries for the deprotonated T*-Ag^{+}-A and G*-Ag^{+}-C pairs (see Table S2) obtained by means of Density Functional theory (DFT) at PBE+vdW level.

Initial configurations:

The initial configurations were chosen to sample all possible combinations of the Watson-Crick and Hoogsteen region of the bases. For every configuration we have placed the cation in the region in between the bases. Representations of such configurations are shown in Fig. S15 for the case of the Guanine homo-duplex (the same approach was used for all other base combinations). If the repulsive forces in the initial configuration are too high large distortions can occur quickly. This problem is typically solved with a very small step size that increases the total time of simulation. Instead, we have performed small translations or rotations on the initial configurations around the indicated position in Fig. S15 that decrease the positive repulsion before the optimization, which moves the cation closer to a bonding configuration. For example, in the first configuration in Fig. S15, the complex with the Ag^+ cation placed in the middle of the region optimizes very quickly to the ground state of the bridged Guanine homo-duplex. More interesting is the case when the Ag^+ cation is placed nearest the Oxygen (for the right base), where the left base shifted downwards to decrease the repulsion with the Hydrogen atom, then an optimization with the BFGS algorithm finds the same ground state (global minimum) after only 206 steps.

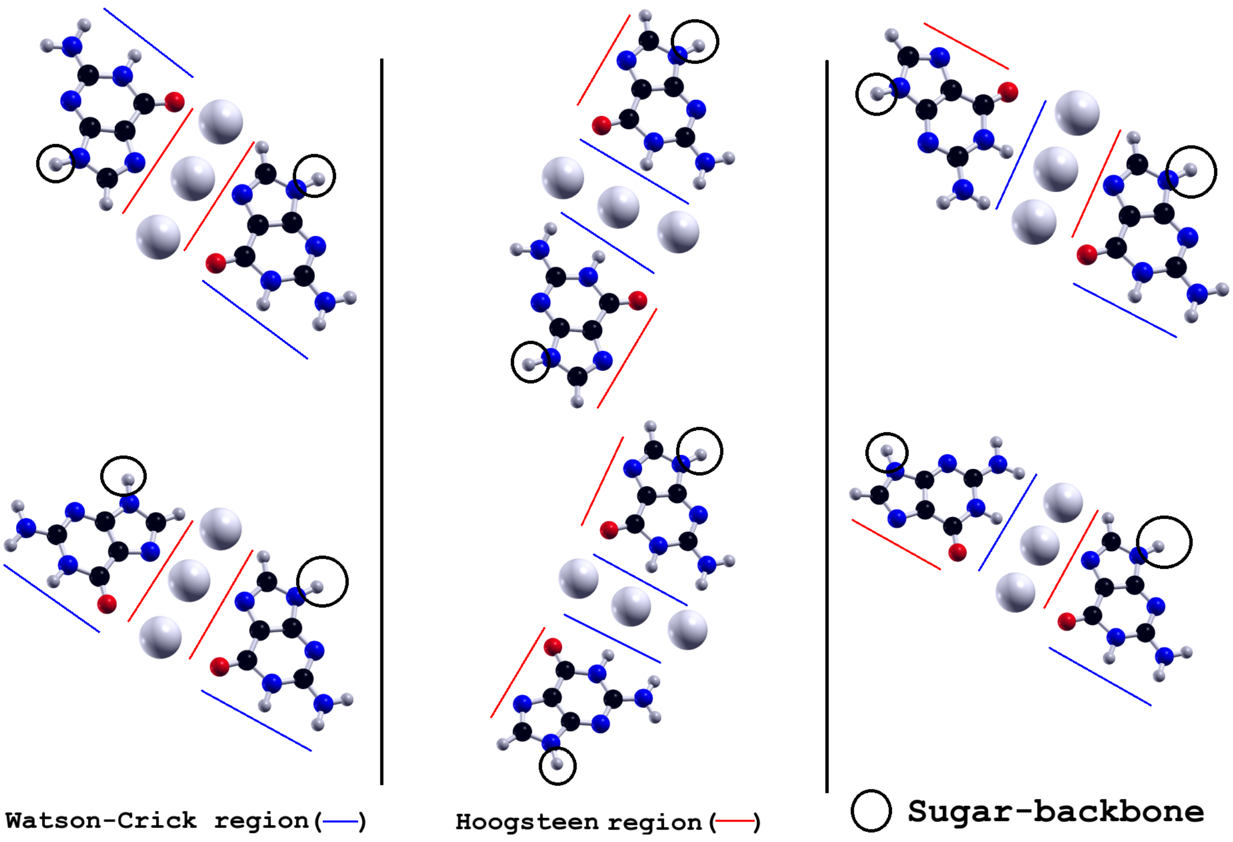

Figure S15) Schematic representation of the initial configurations used for the search algorithm in the case of the Ag^+ homo-duplex with Guanine. The bases are facing their Watson-Crick and Hoogsteen regions and the cation is placed in positions sampling the available bonding space. A small rotation or translation of the bases to decrease high positive repulsions was performed in every case.

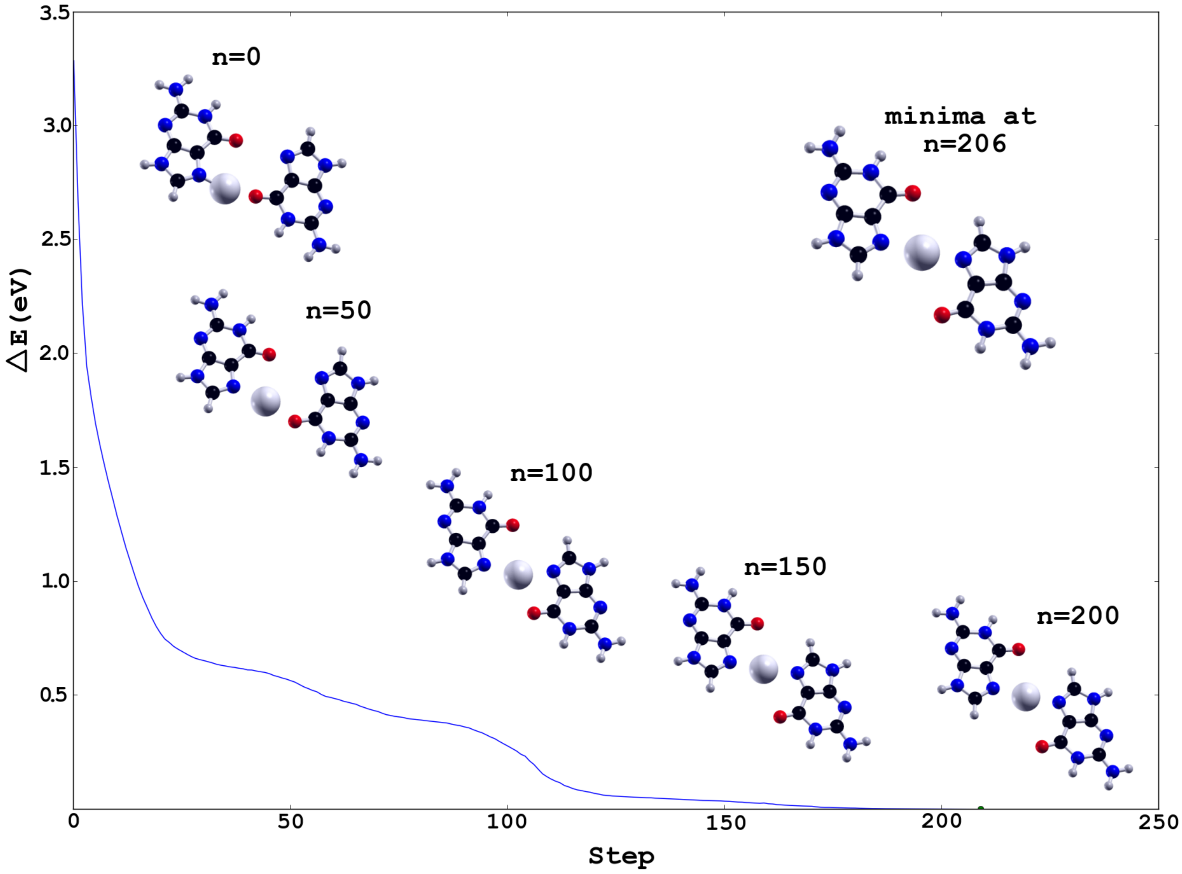

Figure S16) Example of a single force optimization leading to the Ag^{+}-bridged Guanine homo-duplex ground state.
